# Case report: Successful anesthesia management of noncardiac surgery in a patient with single atrium

**DOI:** 10.3389/fphar.2024.1370263

**Published:** 2024-05-01

**Authors:** Hong Cao, Mengmeng Jiang, Zhao Zhuang, Shoushi Wang, Qianqian Cao

**Affiliations:** ^1^ Department of Anesthesia and Perioperative Medicine, Qingdao Central Hospital, University of Health and Rehabilitation Sciences (Qingdao Central Hospital), Qingdao, Shandong, China; ^2^ Department of General Medicine, Qingdao Central Hospital, University of Health and Rehabilitation Sciences (Qingdao Central Hospital), Qingdao, Shandong, China

**Keywords:** single atrium, transthoracic echocardiography, epidural anesthesia, pulmonary hypertension, case report

## Abstract

**Background:**

Single atrium is very rare congenital cardiac anomaly in adults. The prognosis of patients with single atrium is very poor, with 50% of patients dying owing to cardiopulmonary complications in childhood. Herein, we focused on anesthesia management for noncardiac surgery in patients with single atrium.

**Case presentation:**

A 58-year-old male with a history of bilateral varicocele underwent laparotomy for high-position ligation of the spermatic vein. The patient also had a history of single atrium, atrial fibrillation, chronic heart failure, pulmonary hypertension (PH), and complete right bundle branch block (CRBBB). Given the significant complications associated with general anesthesia in patients with PH, we preferred to use low-dose epidural anesthesia for this patient. Transthoracic echocardiography was used to assess cardiac function before and during surgery and guide perioperative fluid therapy. To limit the stress response, we used a regional nerve block for reducing postoperative pain. Furthermore, we used norepinephrine to appropriately increase the systemic vascular resistance in response to the reduction of systemic vascular resistance caused by epidural anesthesia.

**Conclusion:**

Low-dose epidural anesthesia can be safely used in patients with single atrium and PH. The use of perioperative transthoracic echocardiography is helpful in guiding fluid therapy and effectively assessing the cardiac structure and function of patients. Prophylactic administration of norepinephrine before epidural injection may make it easier to maintain the patient’s BP.

## 1 Background

Single atrium is very rare congenital cardiac anomaly in adults with no evidence of atrial septum. The absence of atrial septal structure and the formation of a single heart chamber in the left atrium and right atrium are the main features observed on single atrium echocardiography. Due to the absence of an atrial septal barrier in a single atrium, a large amount of left-to-right shunt blood flow is generated at the atrial level, resulting in a significant overload of right ventricular volume and causing PH in the early stage of the disease. Herein, we report a case of a patient with single atrium, atrial fibrillation, chronic heart failure, and CRBBB who underwent noncardiac surgery. The successful implementation of noncardiac surgery in adult with single atrial not only depends on the choice of anesthesia mode, but also the evaluation of perioperative cardiac function, perioperative monitoring, use of vasoactive drugs, and selection of postoperative analgesia mode. The key to anesthesia is attempting to maintain a balance between pulmonary and systemic circulation resistance. Anesthesiologists should avoid large fluctuations in preload, afterload, and heart rate as much as possible. The aim should be to reduce right-to-left shunt in the atrium, increase arterial oxygen content in the systemic circulation, and prevent heart failure. Low-dose epidural anesthesia has the advantages of slow action, easy control of anesthesia plane, complete analgesia, and good muscle relaxation effect, which is more suitable for such patients than general anesthesia. Considering the complications of general anesthesia in such patients, we selected low-dose epidural anesthesia. Perioperative transthoracic echocardiography was used to guide fluid therapy and noninvasively and effectively assess the cardiac structure and function of the patient.

## 2 Case presentation

A 58-year-old male (height: 160 cm, weight: 68 kg) was scheduled to undergo laparotomy for high-position ligation of the spermatic vein because of bilateral varicocele. The patient had a history of single atrium, atrial fibrillation, pulmonary arterial hypertension, and CRBBB. The patient had been taking digoxin, spironolactone, and furosemide tablets orally for a long time. Physical examination revealed systolic murmurs in the mitral and tricuspid auscultation area, accentuation of the pulmonary valve second heart sound, labial cyanosis, and clubbing of fingers. His vital signs were as follows: temperature, 36.5°C; ventricular rate, 73 beats/min; blood pressure (BP), 112/69 mmHg; and respiratory rate, 19 breaths/min. Under nasal catheterization, oxygen saturation varied between 83% and 95%. If the patient has wheezing after activity, it can be improved after rest. Arterial blood gas analysis under nasal catheterization showed an oxygen partial pressure of 63 mmHg. The laboratory test results were as follows: B-type natriuretic peptide, 850.12 pg/mL; N-terminal b-type natriuretic peptide precursor, 2,460 pg/mL; troponin I, 0.047 ng/kg; and creatine kinase-MB, 31.4U/L.Electrocardiogram showed atrial fibrillation, CRBBB, and change in ST-T. Echocardiography showed single atrium, enlargement of the left and right ventricles, dilation of the pulmonary artery and aorta, severe mitral and tricuspid regurgitation, mild pulmonary valve regurgitation, pulmonary artery pressure (PAP) of 69 mmHg, decrease in left ventricular systolic and diastolic function, and pericardial effusion (medium). Computed tomography showed enlargement of the heart. The preoperative diagnoses were as follows: bilateral varicocele, single atrium, atrial fibrillation, chronic heart failure, PH, and CRBBB.

**FIGURE 1:**
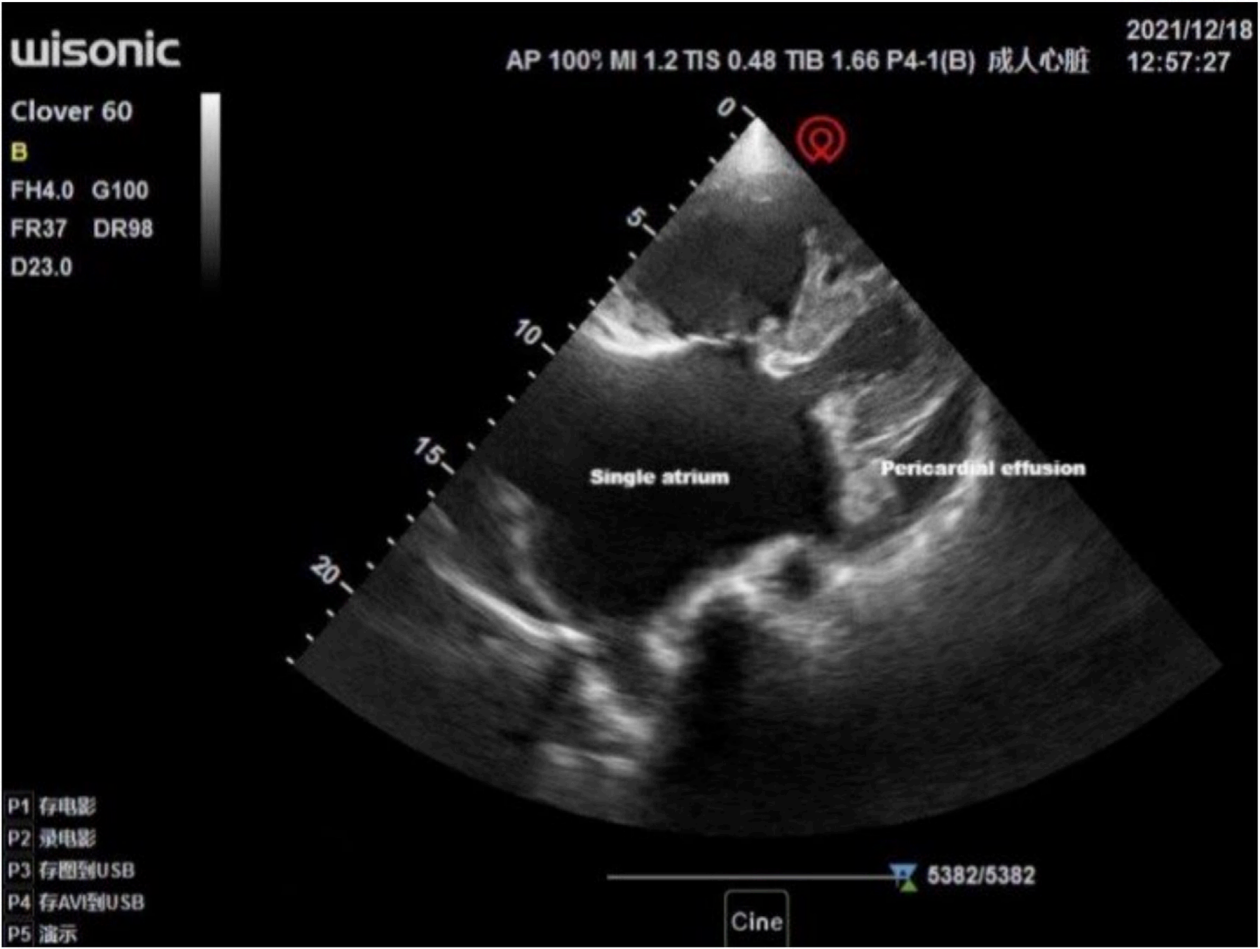
Visualization of the heart by standard transthoracic echocardiography was optimal from the apical views allowing to display a single atrium and moderate amount of pericardial effusion.

**FIGURE 2:**
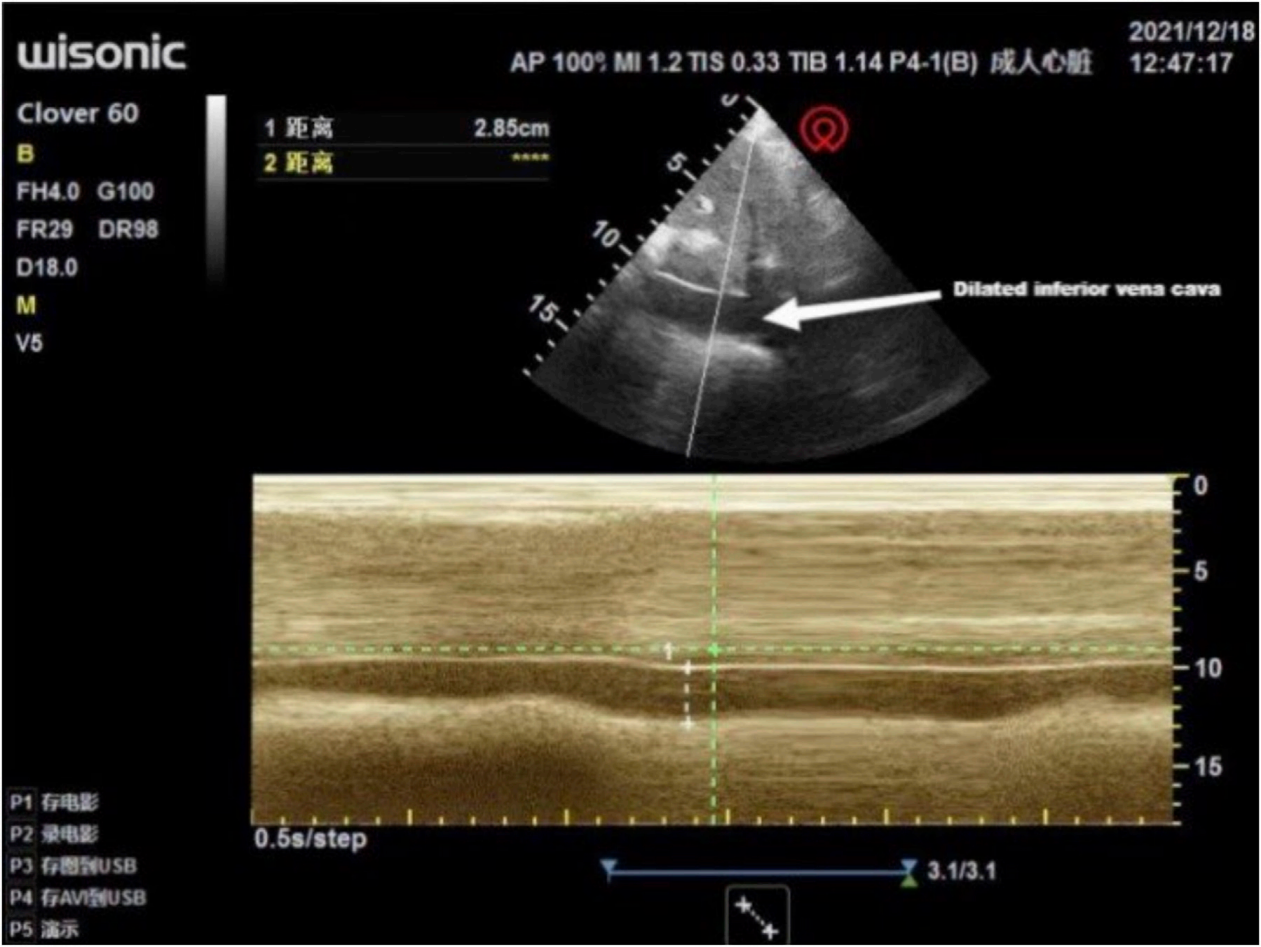
Transthoracic echocardiography showed a dilated inferior vena cava: 2.85 cm in diameter.

**FIGURE 3:**
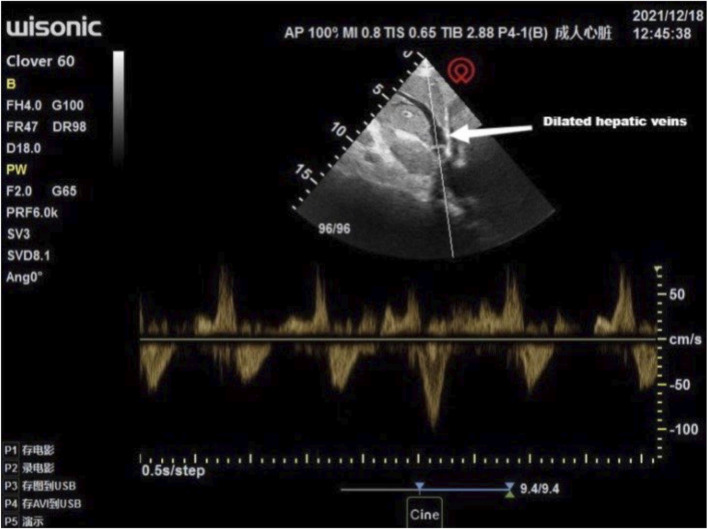
Transthoracic echocardiography showed a dilated hepatic vein.

**FIGURE 4:**
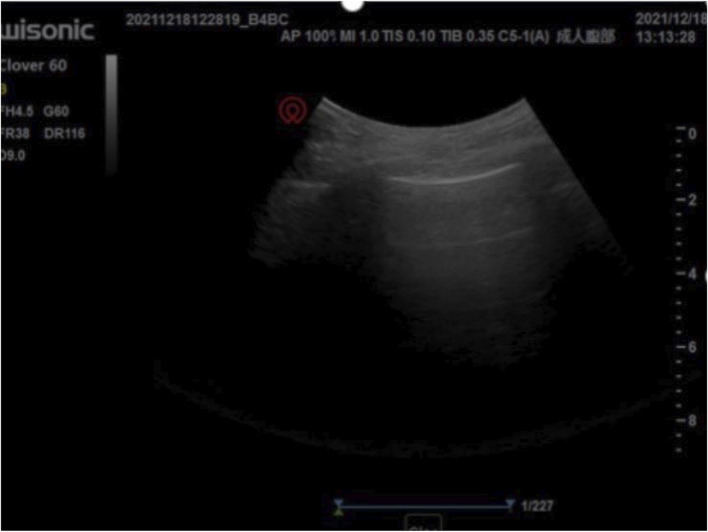
There were no obvious signs of increased extravascular lung water preoperatively. No B-line was found in preoperative and intraoperative lung ultrasonography, and only A-lines were found.

Multiple interdisciplinary meetings were held to discuss the surgery and anesthetic plan. The patient’s New York Heart Association grade was Ⅳ. Oxygen inhalation, cardiotonic, and diuretic therapy were continued. The anesthesiologist used transthoracic echocardiography to assess the cardiac structure and function preoperatively. The heart was optimally visualized from the apical views using standard transthoracic echocardiography, allowing the display of a complete lack of any atrial septal tissue, enlargement of the heart, and decreased left ventricular systolic and diastolic function (Figure 1). In this patient with chronic heart failure, dilated inferior vena cava and hepatic veins suggested chronically high intra-atrial pressure (Figures 2, 3). Due to the changes in the caused by high atrial pressure in this patient, the diameter of the inferior vena cava and respiratory variability could not be correctly used to guide fluid therapy. We could monitor the extravascular lung water (EVLW) of the patient to guide fluid therapy. There were no obvious signs of increased EVLW (Figure 4), indicating that the current amount of fluid therapy was balanced. The apical views showed severe mitral and tricuspid regurgitation (Supplementary Figure S1), and the estimated PAP was 75 mmHg. The patient had tolerated hypoxia for a long time, and the left ventricular function was still compensable; thus, we scheduled laparotomy for high-position ligation of the spermatic vein. Low-dose epidural anesthesia was planned for the patient simultaneously with a regional nerve block, which was used for postoperative analgesia.

Routine monitoring and invasive arterial monitoring were established, including invasive BP, heart rate, oxygen saturation, and electrocardiography. Arterial blood gas analysis while breathing room air revealed an oxygen partial pressure of 59 mmHg. The vital signs of the patient were as follows: ventricular rate, 80 beats/min; invasive BP, 125/66 mmHg; and respiratory rate, 18 breaths/min. The oxygen saturation was 80% while breathing room air and 94% under an oxygen mask. Transthoracic echocardiography was used to reassess the cardiac function and EVLW, and no significant difference was observed in both compared with the preoperative measurements. Fractionation method dosage and low-dose epidural anesthesia were administered in this patient. He was placed in the left lateral decubitus position, and the L2-L3 interspace was chosen as the puncture point for epidural anesthesia. The epidural puncture was performed after administering local anesthesia with 3 mL 1% lidocaine; the puncture process was smooth. A 22-gauge epidural catheter was cephalically inserted into the epidural space, and the length was 3 cm. Before administering drugs through the catheter, the patient was placed in the supine position. Then, 3 mL of 2% lidocaine (Gerheuser and Roth, 2007) was first administered as the experimental dose through an epidural catheter, and the anesthesia level was moved to the L2-L3 interspace after 5 min. Next, 7 mL of 0.375% ropivacaine was slowly given through the administered epidural catheter, followed by left ilioinguinal nerve block with 0.25% ropivacaine 10 mL and left transverse fascia nerve block with 0.25% ropivacaine 10 mL. Bilateral T8 sensory level was obtained by blunt pinprick 25 min later. The patient’s oxygen saturation dropped from 94% to 86% and BP dropped to 91/60 mmHg. Norepinephrine was intravenously pumped at 0.05–0.15 μg/kg/min to maintain BP at 105-125/59-71 mmHg. To avoid anxiety in the patient, low-dose dexmedetomidine was continuously pumped intravenously. The end-tidal partial pressure of carbon dioxide was monitored, which was convenient for observing the patient’s spontaneous breathing. Intraoperative oxygen saturation ranges from 84% to 94%; arterial blood gas analysis showed an oxygen partial pressure of 60 mmHg and a PAP of 74 mmHg. The results of transthoracic echocardiography showed no decrease in cardiac function and no increase in PAP during surgery. The surgery was completed uneventfully without any pain or discomfort, and no drugs were added through the epidural catheter. No formal fluid preload was noted; however, a slow infusion of sodium lactate Ringer’s injection was initiated. Dynamic monitoring transthoracic echocardiography to guide fluid therapy. There were no obvious signs of increased EVLW intraoperatively (Supplementary Figure S2). A total of 400 mL of sodium lactate Ringer’s injection was administered during surgery. The patient sustained intravenous pumping of norepinephrine at 0.15 μg/kg/min to maintain a blood pressure of about 110/60 mmHg and was transferred to the postanesthesia care unit for 1 h and returned to the urological ward. Then, 4 h postoperatively, norepinephrine decreased to 0.02 μg/kg/min and was stopped 9 h postoperatively later with continued oxygen saturation levels of 88%–92% and systolic pressures of 95-107 mmHg. No acute heart failure or dyspnea occurred perioperatively. He was discharged from the hospital 5 days later; he underwent cardiac orthopedic surgery at a follow-up visit 6 months later and had a good recovery. Due to the privacy, we did not get the specific cardiac plastic surgery method of the patient.

## 3 Discussion and conclusion

Single or common atrium, first reported in 1907, is considered to be a form of an extremely rare endocardial cushion defect in adults characterized by a lack of atrial septal tissue (Young and Robinson, 1907). The pathophysiology and clinical features of a single atrium are similar to those of a giant atrial septal defect. In patients with normal PAP, the left-to-right shunt is the main shunt. With an increase in PAP, the pulmonary circulation resistance is greater than the systemic circulation resistance, resulting in right-to-left shunt and Eisenmenger syndrome (Bharwani and Macarthur, 2014). Such patients may have palpitation, shortness of breath, developmental delay, and other manifestations. With the development of the disease, there may be cyanosis, clubbing of fingers and toes, and ejection murmur in the pulmonary valve region. In the late stage of the disease, congestive heart failure, jugular vein distention, and liver enlargement will develop. We described an adult patient with a congenital single atrium, which resulted in severe PH. The preoperative evaluation showed that the patient had tolerated hypoxia for a long time and the left ventricular function was still compensated. According to the present patient’s symptoms, laboratory examination and imaging examination, the patient’s liver and kidney function did not have serious damage. New York Heart Association grade was Ⅳ and pulmonary hypertension suggest a higher risk of anesthesia. The key to anesthesia is attempting to maintain a balance between pulmonary and systemic circulation resistance in patients with single atrium undergoing noncardiac surgery. Maintaining a certain systemic circulation resistance and reducing pulmonary circulation resistance is vital to avoid right-to-left shunt deterioration. Anesthesiologists should avoid large fluctuations in preload, afterload, and heart rate as much as possible to prevent heart failure. Anesthesia is aimed at maintaining cardiac output without worsening the intracardiac shunt.

### 3.1 Anesthetic method

Given the significant complications associated with general anesthesia in patients with PH, low-dose epidural anesthesia was chosen for our patient. In general anesthesia, tracheal intubation can lead to hemodynamic instability owing to the effects of sedation, hypoxemia, sympathetic stimulation, changes in intrathoracic pressure, and hypoventilation (Price et al., 2021). A high plateau pressure can increase the pulmonary vascular resistance (PVR) and affect the right ventricular function.

Most general anesthesia drugs can inhibit myocardial contractility and reduce peripheral vascular resistance, which is manifested as decreased blood pressure and slowed heart rate, resulting in increased right-to-left shunt, aggravating systemic hypoxia symptoms, reducing coronary blood flow, and increasing the risk of cardiac arrest caused by heart failure. General anesthesia during endotracheal intubation may also cause arrhythmia or cardiac arrest due to vagal reflex action. General anesthetics can inhibit cardiac function, reduce venous return and systemic circulation resistance, lead to increased right-to-left shunt, aggravate systemic circulation hypoxia symptoms, reduce coronary blood flow, and increase the risk of cardiac arrest due to heart failure. Spinal anesthesia can lead to rapid sympathetic block, resulting in hemodynamic instability and worsening shunt. The anesthetic plane of epidural anesthesia can be easily controlled, and the hemodynamic effects can be minimized. Because of its slow onset, epidural anesthesia reduces the chances of precipitous hemodynamic changes (Pilkington et al., 2015). However, epidural anesthesia may have an incomplete anesthetic effect. Painful stimuli can cause sympathetic nerve excitation in patients, increasing the chance of switching to general anesthesia. Epidural anesthesia can also reduce systemic circulation resistance, but not to the same extent as that of general anesthesia. Furthermore, the hemorrhagic risk of venepuncture in the spinal canal may be exacerbated by intraspinal venous rage due to chronic venous hypertension (Connors et al., 1996). In this case, there was a small decrease in oxygen saturation and BP after epidural anesthesia, indicating a decrease in systemic circulation resistance after anesthesia that resulted in a worsening of the right-to-left shunt; however, it was still within the tolerance limit of the patient.

### 3.2 Perioperative monitoring

The establishment of invasive arterial blood pressure before anesthesia allows continuous assessment of coronary pulsation perfusion pressure and predetermination of systemic diastolic blood pressure targets. Central venous pressure (CVP) monitoring can be used to assess changes in left ventricular preload, left ventricular charging pressure, and overall left ventricular performance. Perioperative transesophageal echocardiography can assess ventricular pulsation function, ventricular filling, dynamic tricuspid regurgitation, and pulmonary artery pressure, but requires general anesthesia.

Perioperative transthoracic echocardiography may be preferred for careful preoperative assessment and perioperative echocardiographic monitoring of such patients, if the mode of anesthesia and type of procedure permits. Echocardiography is aimed at the examination of the heart structure, function and hemodynamics, and its structural examination is mainly to understand the anatomy of the heart and whether the ventricular wall movement is abnormal. In this case, transthoracic echocardiography performed regularly perioperatively facilitated the monitoring of PAP and the effects of fluid therapy. Due to the changes in the caused by high atrial pressure in this patient, the diameter of the inferior vena cava could not be correctly used to guide fluid therapy. We could monitor EVLW to guide fluid therapy. EVLW is composed of alveolar, interstitial, and intracellular fluids. The main components of EVLW are surfactant, extravasated blood plasma, intracellular fluid, and lymphatic fluid (Shyamsundar et al., 2013). Inappropriate fluid management strategies may lead to increased EVLW, worsening pulmonary edema, and even increased mortality (Ingelse et al., 2016). The dynamic monitoring of EVLW is the basis for providing appropriate volume for critically ill patients to ensure resuscitation and to avoid excessive increase of EVLW. Lung ultrasonography (LUS) is one of the methods used for the evaluation of EVLW (Pirompanich and Wattanathum, 2015). The number of B-lines on LUS can be used to evaluate the degree of EVLW increase (Shyamsundar et al., 2013). In this case, LUS was used to dynamically monitor the EVLW of the patient to provide reasonable fluid therapy. No B-line was found in preoperative and intraoperative LUS, and only A-lines were found, which were present in normal LUS manifestations. There were no obvious signs of increased EVLW, indicating that the current amount of fluid therapy is balanced. The choice of more advanced monitoring methods such as pulmonary artery catheters, transpulmonary dilution, and transesophageal echocardiography depends on local availability and expertise (Price et al., 2021).

### 3.3 Choice of vasoactive drugs

To decrease the afterload of the right ventricle, levosimendan and milrinone are recommended as selective pulmonary vasodilators because they reduce PAP but not systemic circulation resistance and MAP (Kundra et al., 2018). Milrinone is a selective phosphodiesterase Ⅲ inhibitor, which enhances myocardial contractility and reduces systemic and pulmonary circulation resistance by inhibiting cAMP decomposition and enhancing protein kinase A activity. Levosimendan is a calcium sensitizer, which can inhibit phosphodiesterase Ⅲ, improve left ventricular function, dilate pulmonary vessels, and reduce PAP and PVR. Patients with low systemic vascular resistance may need additional vasopressor treatment. Norepinephrine and vasopressin are the preferred medications. Maintaining blood pressure is essential to ensure coronary perfusion and the right cardiac output. Hypotension caused by reduced systemic circulation resistance is often treated with norepinephrine and vasopressin. Levosimendan, milrinone, dobutamine, and other inotropic drugs can effectively improve the systolic and diastolic function of the heart and ensure the stability of the overall hemodynamics; however, the vasodilatation effect may need to be combined with norepinephrine and vasopressin (Abdelazziz and Abdelhamid, 2019; Kanemaru et al., 2020).

Norepinephrine is a first-line treatment in most centers and improves coronary blood flow in right heart failure models. Low-dose norepinephrine infusion can increase systemic circulation resistance, reduce right-to-left shunt, increase pulmonary blood, and maintain systemic blood flow balance. In our patient, to correct the reduced systemic circulation resistance caused by epidural anesthesia, continuous pumping of a low-dose norepinephrine was effective and improved hypoxemia and hypotension of the patient. Prophylactic administration of norepinephrine before epidural injection may make it easier to maintain the patient’s BP.

### 3.4 Postoperative analgesia

Adequate administration of analgesia is essential. Pain stimulation can activate the sympathetic nervous system to release catecholamines and increase heart rate, myocardial oxygen consumption, and PVR, with rapid onset of acute right ventricular decompensation and cardiac arrest. Perfect postoperative analgesia can reduce postoperative inflammatory stress response, enhance postoperative immune function, reduce postoperative cognitive dysfunction and other advantages, which is conducive to the accelerated recovery of patients after surgery. There should be adequate postoperative analgesia, using multimode analgesia, but to avoid excessive sedation caused to respiratory function depression. In our patient, epidural anesthesia combined with a regional nerve block maintained optimal pulmonary and systemic vascular resistance intraoperatively. Regional nerve block analgesia can last for 12 h and efficiently restrain the stress response of postoperative pain.

In conclusion, multiple interdisciplinary meetings are necessary for patients with single atrium undergoing noncardiac surgery. Anesthesiologists must understand the pathologic damage to the cardiovascular system and the corresponding pathophysiological changes and determine the hemodynamic targets. Keeping a balance of pulmonary circulation and systemic circulation resistance is the key to anesthesia management. Perioperative echocardiography should be used to monitor cardiac function and guide fluid therapy. Appropriate anesthesia method and anesthetics drugs should be used to minimize the effect of anesthesia on circulation function.

## Data Availability

The original contributions presented in the study are included in the article/Supplementary Material, further inquiries can be directed to the corresponding author.

## Abbreviations

BP, blood pressure; CRBBB, complete right bundle branch block; EVLW, extravascular lung water; LUS, lung ultrasonography; PH, pulmonary hypertension; PAP, pulmonary artery pressure; PVR, pulmonary vascular resistance.

## References

[B1] Abdelazziz M. M. Abdelhamid H. M. (2019). Terlipressin versus norepinephrine to prevent milrinone-induced systemic vascular hypotension in cardiac surgery patient with pulmonary hypertension. Ann. Card. Anaesth. 22 (2), 136–142. 10.4103/aca.ACA_83_18 30971593 PMC6489405

[B2] Bharwani F. Macarthur A. (2014). Review of a high-risk obstetric anesthesia antepartum consult clinic. Can. J. Anaesth. 61 (3), 282–283. 10.1007/s12630-013-0094-5 24347354

[B3] Connors A. F. Speroff T. Dawson N. V. Thomas C. Harrell F. E. Wagner D. (1996). The effectiveness of right heart catheterization in the initial care of critically ill patients. SUPPORT Investigators. JAMA 276, 889–897. 10.1001/jama.276.11.889 8782638

[B4] Gerheuser F. Roth A. (2007). Epidural anesthesia. Anaesthesist 56 (5), 499–523. 10.1007/s00101-007-1181-1 17431551

[B5] Ingelse S. A. Wösten-van Asperen R. M. Lemson J. Daams J. G. Bem R. A. van Woensel J. B. (2016). Pediatric acute respiratory distress syndrome: fluid management in the PICU. Front. Pediatr. 4, 21. 10.3389/fped.2016.00021 27047904 PMC4800174

[B6] Kanemaru E. Yoshitani K. Kato S. Fujii T. Tsukinaga A. Ohnishi Y. (2020). Comparison of right ventricular function between patients with and without pulmonary hypertension owing to left-sided heart disease: assessment based on right ventricular pressure-volume curves. J. Cardiothorac. Vasc. Anesth. 34 (1), 143–150. 10.1053/j.jvca.2019.05.025 31227379

[B7] Kundra T. S. Prabhakar V. Kaur P. Manjunatha N. Gandham R. (2018). The effect of inhaled milrinone versus inhaled levosimendan in pulmonary hypertension patients undergoing mitral valve surgery - a pilot randomized double-blind study. J. Cardiothorac. Vasc. Anesth. 32 (5), 2123–2129. 10.1053/j.jvca.2018.04.022 30098861

[B8] Pilkington S. A. Taboada D. Martinez G. (2015). Pulmonary hypertension and its management in patients undergoing non-cardiac surgery. Anaesthesia 70 (1), 56–70. 10.1111/anae.12831 25267493

[B9] Pirompanich P. Wattanathum A. (2015). Ultrasound assessment for extravascular lung water in patients with septic shock. Crit. Care 19 (1), 223. 10.1186/cc14303 25944130

[B10] Price L. C. Martinez G. Brame A. Pickworth T. Samaranayake C. Alexander D. (2021). Perioperative management of patients with pulmonary hypertension undergoing non-cardiothoracic, non-obstetric surgery: a systematic review and expert consensus statement. Br. J. Anaesth. 126 (4), 774–790. 10.1016/j.bja.2021.01.005 33612249

[B11] Shyamsundar M. Attwood B. Keating L. Walden A. P. (2013). Clinical review: the role of ultrasound in estimating extra-vascular lung water. Crit. Care 17 (5), 237. 10.1186/cc12710 24041261 PMC4057491

[B12] Young A. H. Robinson A. (1907). Some malformations of the human heart. Med. Chron. 47, 96–106.

