# Supplementary figures and images for: Case report: Successful anesthesia management of noncardiac surgery in a patient with single atrium
Source: Front Pharmacol. 2024 May 1;15:1370263. doi: 10.3389/fphar.2024.1370263 (PMC11097672; doi:10.3389/fphar.2024.1370263)

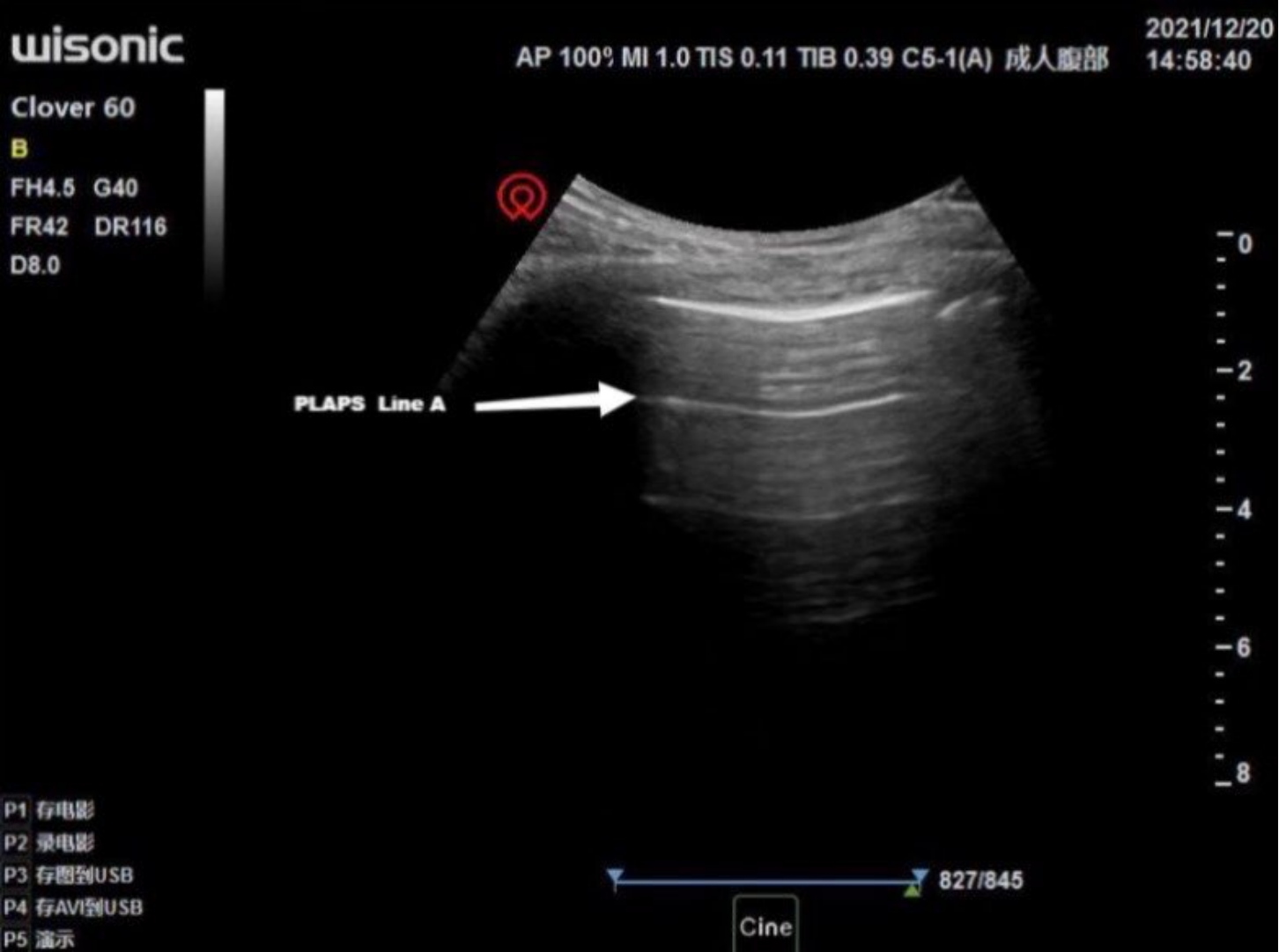

Supplement: Supplementary file 1 [file Image2.TIF]

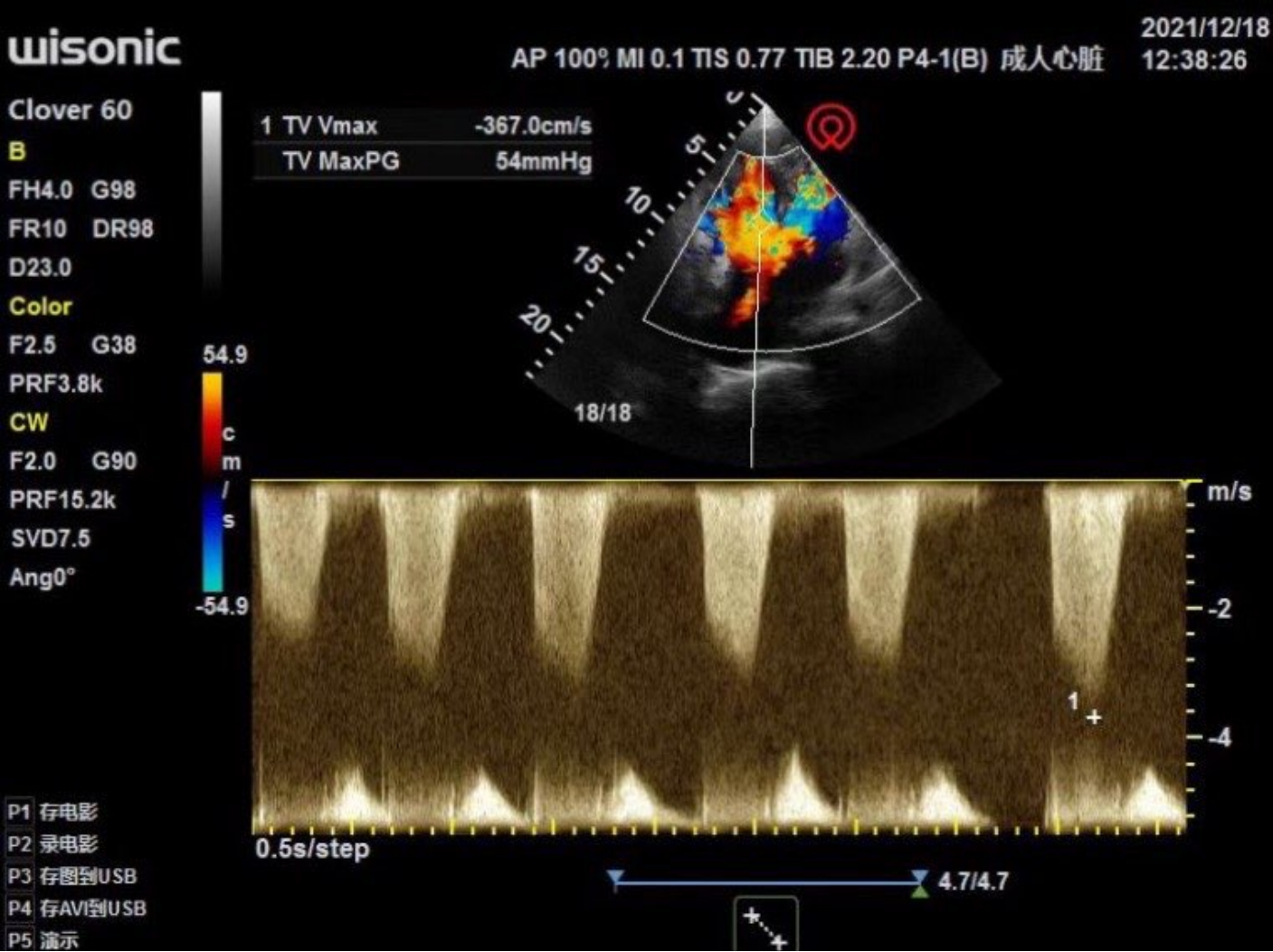

Supplement: Supplementary file 2 [file Image1.TIF]
